# Light People: Professor Xi-Cheng Zhang

**DOI:** 10.1038/s41377-022-00955-w

**Published:** 2022-09-14

**Authors:** Hui Wang

**Affiliations:** Changchun Institute of Optics, Fine Mechanics and Physics, Chinese Academy of Sciences, Changchun, China

**Keywords:** Laser-produced plasmas, Other photonics

## Abstract

As 5 G ever becomes a part of everyday life and 6 G is also on the agenda, terahertz technology has gradually become known by the public, and is referred to as the “third eye” for human beings to understand the world.

Terahertz is considered one of the top ten technologies that will change the world due to its unique properties. In 2019, astronomers were able to observe a black hole for the first time, using a terahertz astronomical telescope. Terahertz is no longer far away from our lives, whether it is information, medicine, or food safety and other fields. It is everywhere, triggering major changes in people’s work and lifestyle.

Today’s guest of Light People is an internationally renowned scholar who has been engaged in terahertz research for many years, Professor Xi-Cheng Zhang works at the University of Rochester, one of the three major optics educational centers in the United States. As a pioneer in the field of terahertz technology, after years of practice, he has successfully generated terahertz waves using water, changing how we see water with THz wave forever.

Next, please follow the Light Science Editor into the terahertz world to learn about the latest and most cutting-edge science and technology and the “story” behind it.

## Abstract (web summary)

Prof. Xi-Cheng Zhang, as a pioneer in the field of terahertz technology, has successfully generated terahertz waves using water, changing how we see water with THz wave forever.

## Graphical abstract

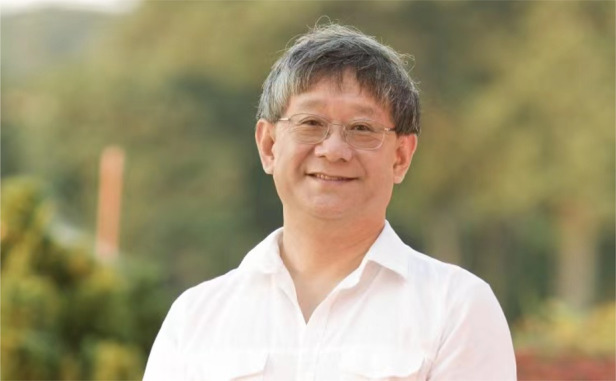

**Biography:** Professor Xi-Cheng Zhang graduated from Peking University in 1982 and received his PhD in physics from Brown University, Providence, RI in 1986. He was a visiting scientist at MIT in 1985; 1985 to 1987, he worked in the Physical Technology Division of Amoco Research Center; 1987 to 1991, he was in the Electrical Engineering Department at Columbia University. Dr. Zhang joined Rensselaer in 1992. He is Parker Givens Chair at The Institute of Optics, University of Rochester. He is a Fellow of AAAS, APS, IEEE, Optica, and SPIE. He is elected foreign member of Russian Academy of Sciences. He has received 29 US patents, authored or co-authored over 350 refereed scientific papers with his h-index of 94. His research interests center around Terahertz waves, also known as T-rays, which exist within a frequency range between microwave and infrared. His research is focused on the generation, detection, and applications of free-space THz beams with ultrafast optics.

**Figure:**
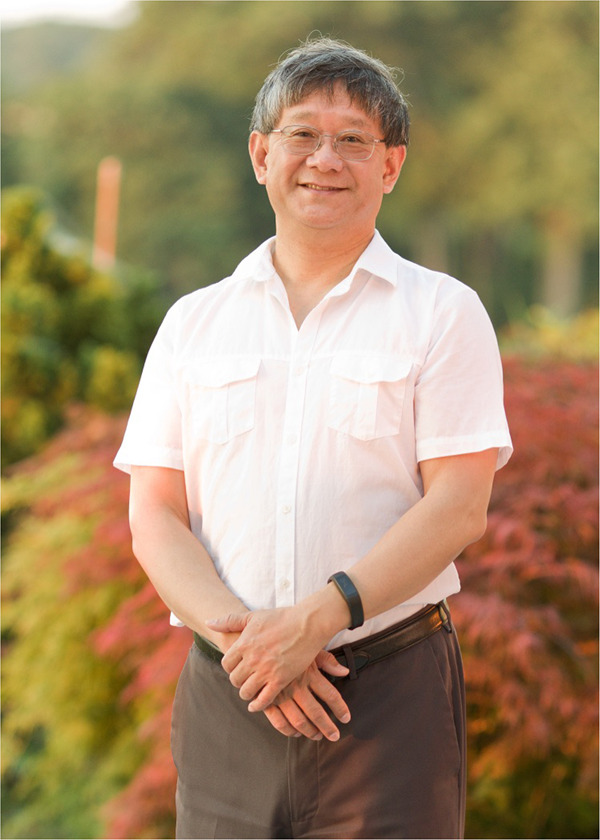

**1. Terahertz science refers to the study of a certain section of electromagnetic waves which can “see through” many things. Due to the lack of detectors and launch sources, the development of terahertz has been lagging behind. In recent years, with the improvement of scientific research methods, many achievements have been made in this field. You are an internationally recognized pioneer in the field of terahertz research, long-term THz imaging and bio-medical applications, as well as ultra-fast photonics, optoelectronics research, can you briefly tell us its development history and future trends?**

Prof. Zhang: In general, the Terahertz (THz) band frequency range is 0.3–10 THz. It is a spectral window with rich scientific opportunities that is, at present, stunted by limited technology. The region has long been considered the last remaining scientific gap in the electromagnetic spectrum, which is underdeveloped but ripe for exploitation. This field shows great promise for a variety of reasons including small-scale electron accelerators, contact-less probes, high-field nonlinear optics, and broadband material characterization. Frequency signature of large molecules at THz band is always interested by the THz community.

**2. With the gradual deepening of terahertz research, the importance of terahertz spectrum and technology in the fields of physics, chemistry, biology, electronics, etc. has also become clear, and it has been used in many aspects of the society and economy, such as: terahertz security inspection equipment, biology Imaging, through-wall radar, etc. Why can terahertz be used in so many different ways? In which areas do you think it will shine in the future?**

Prof. Zhang: THz radiation, which is electromagnetic radiation in a frequency interval from 0.3 to 10 THz (1 mm–30 μm in wavelength), is the next frontier in science and technology. This band occupies a large portion of the electromagnetic spectrum between the infrared and microwave bands. Basic research, new initiatives, and developments in advanced sensing and imaging technology with regards to the THz band remain unexplored compared with the relatively well-developed science and technology in the microwave and optical frequencies.

Historically, THz technologies were used mainly within the astronomy community for studying the background of cosmic far-infrared radiation, and by the laser-fusion community for the diagnostics of plasmas. Since the first demonstration of THz wave time-domain spectroscopy in the late 1980’s, there has been a series of significant advances (particularly in recent years) as more intense THz sources and higher sensitivity detectors provide new opportunities for understanding the basic science in the THz frequency range.

As developments move forward, THz science will not only have an impact on material characterization and identification, it will also have potential applications in the fields of communications, imaging, medical diagnosis, health monitoring, environmental control, and chemical and biological sensing, as well as security and quality control applications. Twenty-first century research in the THz band is one of the most promising areas of study for transformational advances in imaging and other interdisciplinary fields.

The cost of typical THz system is still too high for the large scale industrial applications, especially competing with other low cost methods.

Perhaps the most needed industrial application is non-destructive evaluation for quality control and defect inspection. Approximately 300 companies have contacted me before, and over 80% of these companies expressed their need for non-destructive evaluation. For example, THz radiation can penetrate optical and infrared opaque materials and it has higher spatial resolution than microwave radiations, making it uniquely suitable for a variety of imaging applications ranging from industrial quality control and biomedical applications to security applications.

**3. We know that solids, gases and plasma can be used to produce THz waves, but how did you come up with the idea that liquid water can also produce high-intensity and broadband THz waves using femtosecond laser pulses? What are the advantages of liquid compared to the other three forms of substance?**

Prof. Zhang: It is well known that water, the most common liquid, is a strong absorber in the THz frequency range, therefore liquid water has historically been sworn off as a source for THz radiation.

Normal universal matters are made of four states: solid, liquid, gas, and plasma. The generation of THz wave from solids, gases, and plasmas has been demonstrated, used, and understood for decades. However, the THz wave generation from liquid sources was conspicuously absent, especially from liquid water due to water’s infamously strong absorption characteristics in the THz regime. It is reasonable to expect that liquids might have unique properties if they could be harnessed as THz sources. Liquids have a high molecular density, close to that of solids, meaning that light over a certain area will interact with many more molecules than an equivalent cross-section of gases. This makes liquids very good candidates for the study of high-energy-density plasma. A successful investigation in THz generation from liquids will complete the last piece of the matter-phase puzzle for THz sources. New physics will be developed to fully characterize this process in liquids, especially in water and related materials, to support new THz wave science, technology and applications.

**Figure:**
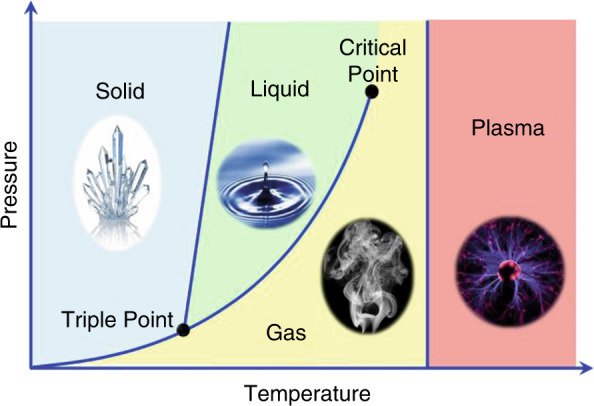
Three of four states, solid, gas, and plasma, have been used to generate THz waves. The use of liquids as THz sources is a real challenge.

**4. Since 1988, you have been engaged in the research of THz imaging and its bio-medical applications, as well as in the fields of ultrafast photonics and optoelectronics. You are also committed to the global promotion and development of terahertz science and technology. Why did you choose this research direction? What do you think of terahertz after spending so many years studying it?**

Prof. Zhang: In the late 1980s, there were three groups in USA actively investigating pulsed THz waves controlled by ultra-short laser: IBM Thomas J. Watson Research Center led by Dan Grischkowsky (just passed away in June this year), Bell Laboratories led by Martin Nuss, and Columbia University led by David Auston. There was no established experimental technique to generate or detect broadband THz waves. They had to fabricate optical components and develop a THz measurement system. They tried to generate THz waves by using a photoconductive antenna (also called an Auston switch) or electro-optic rectification. During my time at Columbia University, I was curious about whether the THz waves have see-through capability. I observed a pulsed THz wave emitted from an unbiased semiconductor wafer under a femtosecond laser beam excitation. I remember that during the initial excitement. I tried many materials: dielectrics, metals, tissues, clothes, lumbers, everything we could find around us, including my fingers, looking for new THz wave emitting materials to understand their physics. Most of us used photoconductive dipole antennas to detect THz waves during the early days. Now they are many different methods to generate THz wave, especially using short pulse lasers.

**Figure:**
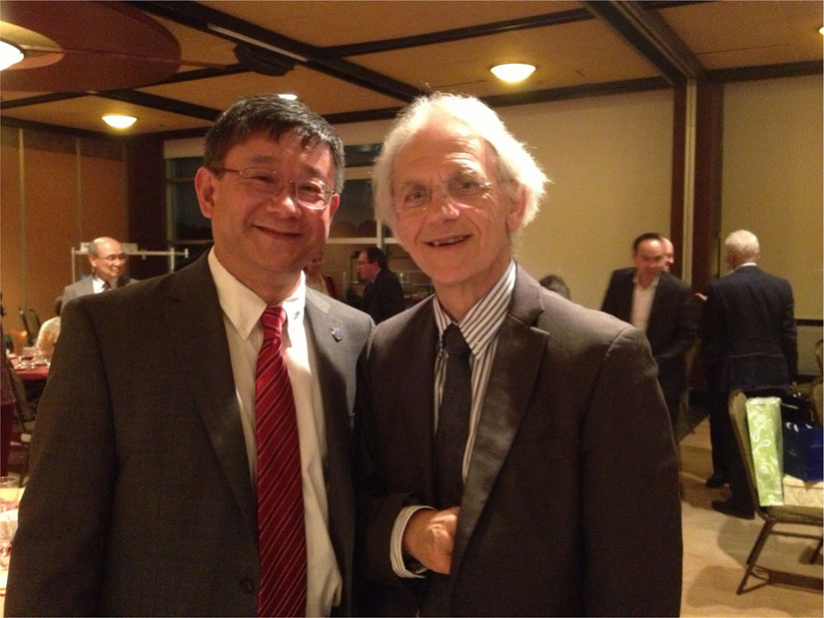
Prof. Xi-Cheng Zhang and Gerald Mourou, 2018 Nobel Laureate in Physics.

**5. In the latest Scholar h index ranking**, ***Light: Science & Applications*** **(Light) was ranked second in the field of optics and photonics in the world. This year also coincides with the** **10**^**th**^ **anniversary of the launch of Light. Do you have any expectations and suggestions as editor-in-chief of Light? Can you share your story with the Light Journal with us?**

Prof. Zhang: After I finished my two-term service as the Editor-in-Chief of *Optics Letters* (2014–2019), I have agreed to take the role as the Executive Editor-in-Chief then co-Editor-in-Chief of Light. Light made impact to optics and photonics community. I am honored to work with truly outstanding editorial team and staffs. Light made amazing achievements in past 10 years, now we are facing more competition, since there are several new optics and photonics journals than 10 years ago. As EiC of Light, I will make sure that Light will continue providing the top service to optics community, attract and publish most important scientific and technology papers.

**Figure:**
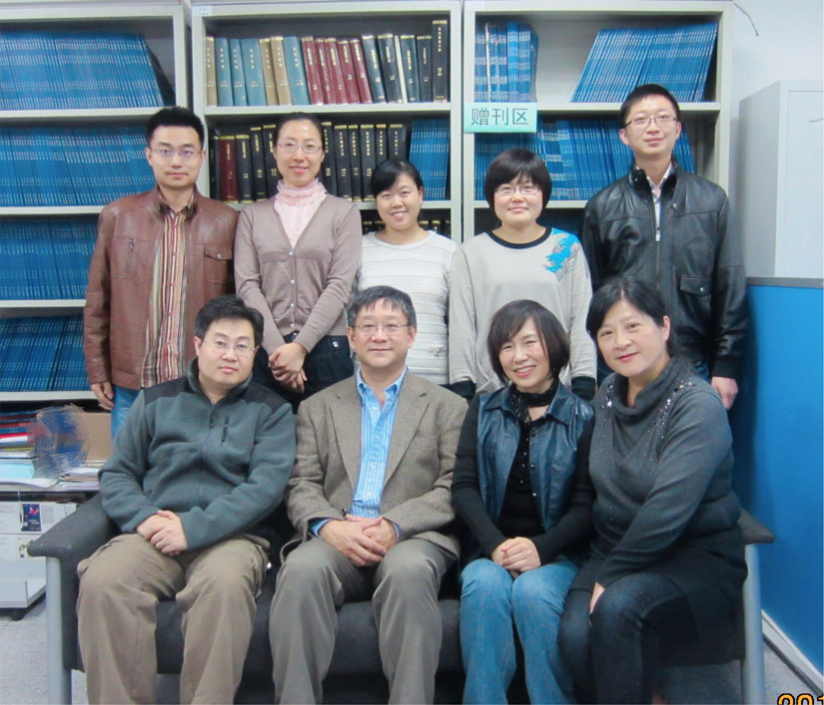
Prof. Xi-Cheng Zhang visited the CIOMP Editorial Department in 2012.

**6. How do you think mentors should train students?**

Prof. Zhang: You should not just simply treat the student training and education as job. It is a passion. You should treat the students like your own kids. Let them grow, give them freedom, trust them, and encourage them when they made mistakes (learn from mistakes is a typical way we grow up anyway).

**Figure:**
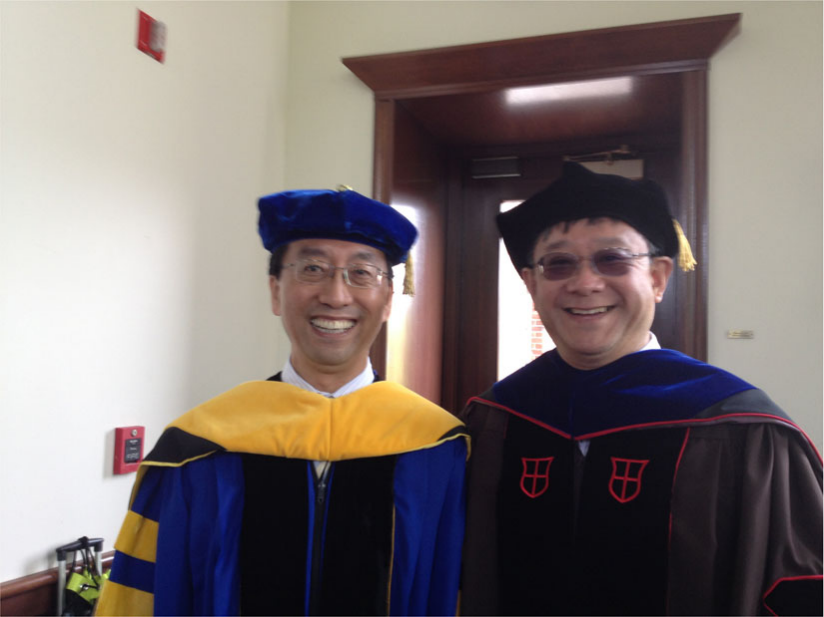
Prof. Xi-Cheng Zhang with Jie Zhang, President of Shanghai Jiaotong University during University of Rochester Commencement in Rochester, New York, 2013.

**7. You and your research group innovatively used pulsed laser pumping to observe the emission of terahertz waves in a flowing water film, and made history. During the experiment, how did you adjust your feelings when you failed? Is there any anecdote you like to share with us?**

Prof. Zhang: About 14 years ago, I suggested students and researchers in my group to consider to try THz wave generation from liquid water using femtosecond lasers. Einstein relation says that if the atom has a strong absorption, then through the Einstein coefficients (A and B, and they are proportional), it should have strong emission as well. Since water molecule has strong absorption, then it could have strong emission. However, we have tried more than 10 years in my lab, we could not demonstrate it. A few years ago I have a student from Huazhong University of Science and Technology. I asked him to try it, after several PhD students and postdoctoral gave this water source project up. We started with thin water film, and flowing water, then using bulk water. The student (his name is Qi Jin), did not see the hope at the beginning, he almost gave it up as well, like his peers in the same group. However, he finally decided to give one or two week more time on it. The day before he planned to give it up, he found the possible signal. Then we refined the experiment, also hired a postdoc, Dr. Yiwen E, from Institute of Physics, Chinese Academy of Sciences (CAS). We finally demonstrated it successfully in 2017. It is the first report in the world from our group in 2017. Now, Dr. Yiwen E is a top researcher in the field of THz liquid photonics.

**8. You are a Fellow or member of AAAS, APS, IEEE, OPTICA and SPIE, elected foreign member of Russian Academy of Sciences, and actively participate in various international Academic organizations. In addition, you are also busy with giving lectures and reports at various research institutes, universities and companies. Why do you take part in these academic public affairs activities? What do you think you have gained from them?**

Prof. Zhang: Yes, I have given more than 500 lectures or talks. I think this is a society service. At certain age and experience, someone should do it for the society. What do I got from these travels? I love good food, I often got very nice meals during the conferences.

In person interaction is always a big plus. One of the drawbacks is time consuming during the travel, and jet lag is another unpleasant thing.

**9. In your eyes, what is the charm of scientific research? What have you gained from your research life?**

Prof. Zhang: It would be boring if I do not do the research. However, I love animals, and enjoy the gardening. New discovery is the happiest time during the research (like harvest).

**10. Who is the person who has had the biggest influence on you so far? Why?**

Prof. Zhang: Career wise: My high school teachers in Beijing 21 Middle School. They are my class teacher (math teacher) and physics teacher. The class teacher’s passion gave me the experience how a teacher teaches, and physics teacher taught us how to understand science.

Personal wise: my father. My father was editor in chief of Chinese News Agency for more than 30 years. I was EiC of several scientific journals now. Of course, my wife now is the most important person in my life.

**11. Do you have any hobbies? How do you balance work and life?**

Prof. Zhang: I love to travel, enjoy good food. I also enjoy gardening. It is good for the health.

**Figure:**
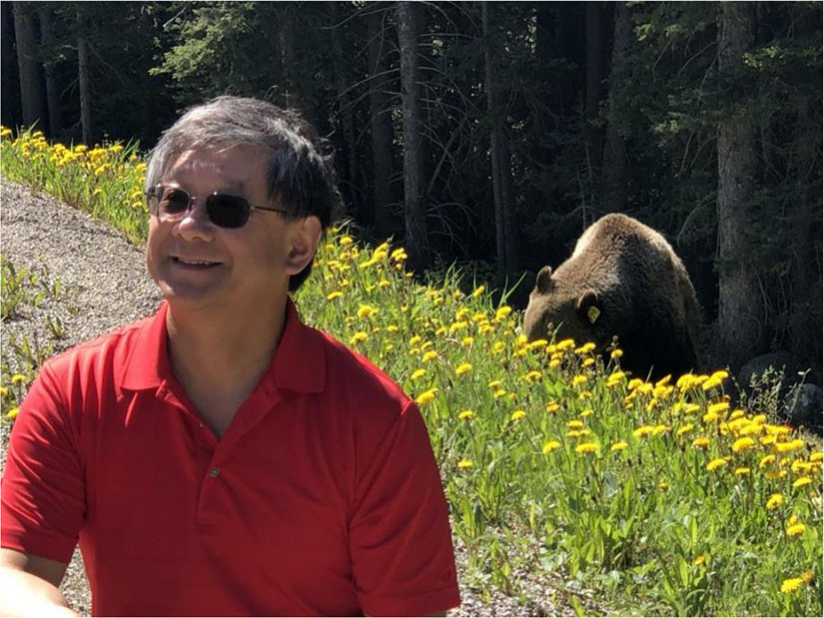
Prof. Xi-Cheng Zhang in Banff National Park, Canada.

**Figure:**
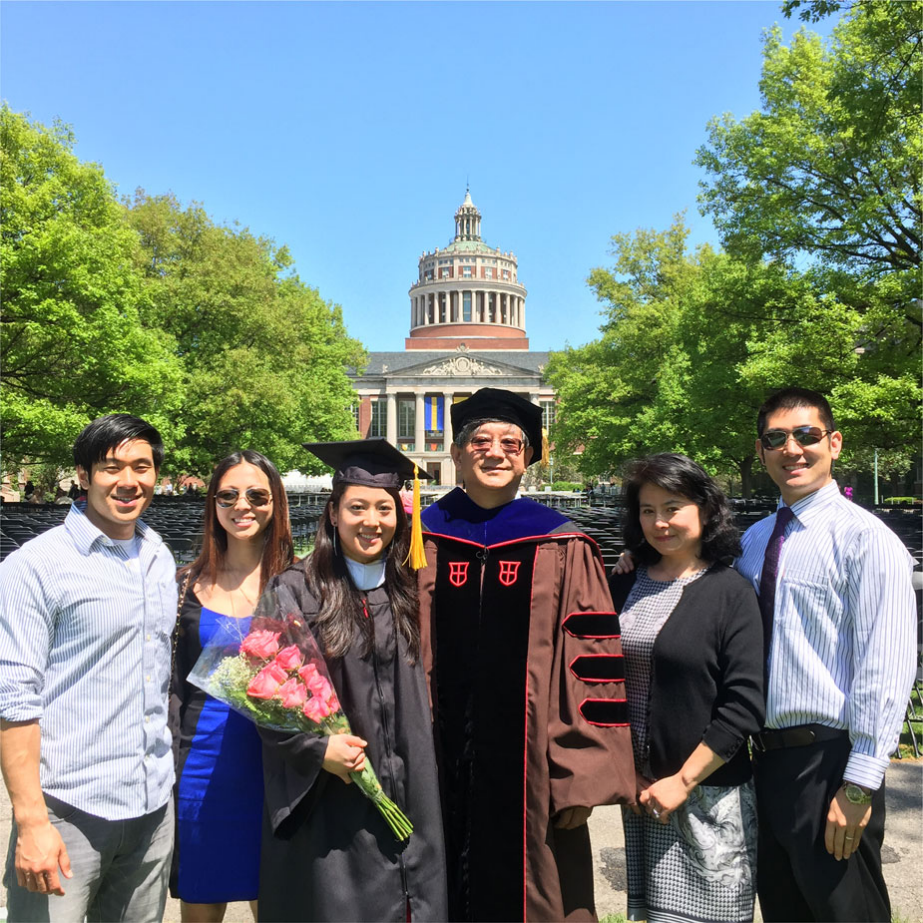
Prof. Zhang’s family participated in his daughter’s graduation ceremony at University of Rochester.

**12. As far as I know, your wife is very supportive of your scientific research career. Can you give some examples? If you could say a sentence here to express your feelings, what would you say to her?**

Prof. Zhang: My wife is the key person encouraging me with bigger picture in my career. “I love you and thank you” is the one sentence for my lifetime feeling for my wife.

**Figure:**
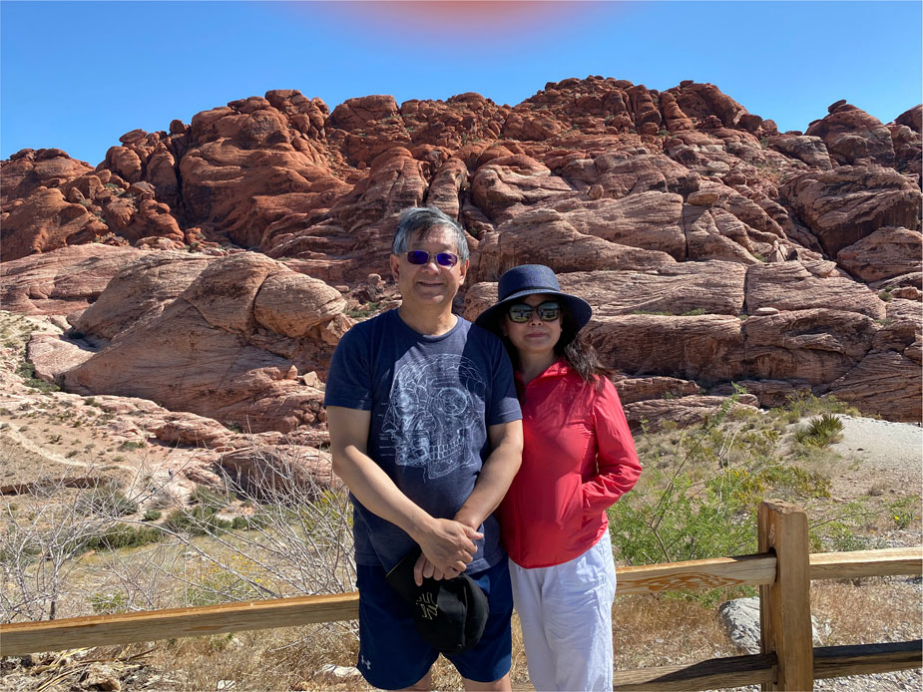
Prof. Zhang and his wife has been married for 40 years, and they have such lovely and memorable time together.

**Figure:**
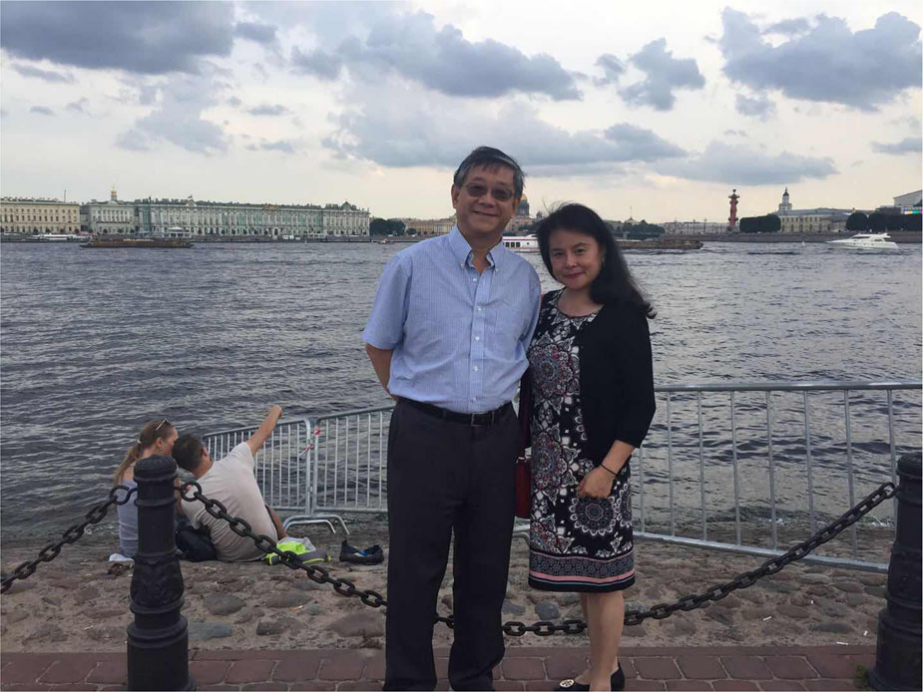
About 10 years, Prof. Zhang and his wife visited St. Petersburg, Russia for white night in June, each year.

**Figure:**
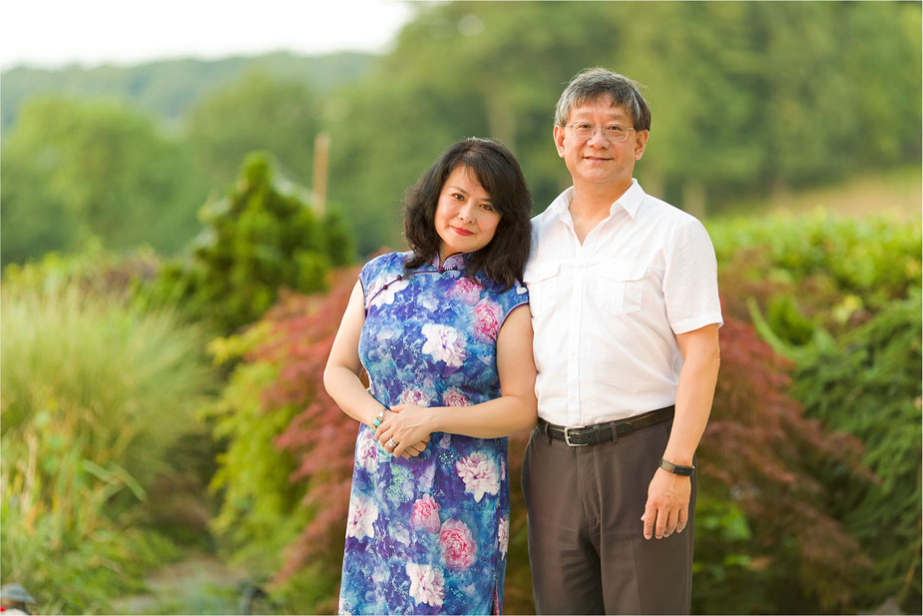
Prof. Zhang and his wife in their backyard.

**13. What advice and expectations do you have for young scientific researchers?**

Prof. Zhang: I often tell my students: you have tried to do something, you might not get it as you wish to get; however, if you did not even try it, you will guarantee not to get it.

**Light special correspondent**

*WANG Hui is the Deputy Director of Division of International Cooperation in the Changchun Institute of Optics, Fine Mechanics and Physics (CIOMP), Chinese Academy of Sciences (CAS). She currently works on international communication and cooperation for the CIOMP and was a founding member of the journal Light: Science & Applications, which is a joint publication of Nature Publishing Group and CIOMP. She has published several articles in Acta Editologica, International Talent, Light: Science & Applications, etc., and was invited to contribute an article to SPIE Women in Optics in 2015. She is the initiator of the Rose in Science event and the co-sponsor and moderator of the iCANX Story. She has interviewed Donna Strickland, Nobel Laureate in Physics; Jean-Marie Lehn, Nobel Laureate in Chemistry; Johanna Stachel, the first female president of the German Physical Society; Chennupati Jagadish, president of the Australian Academy of Sciences; Carmen Menoni, president of the IEEE Photonics Society; Lin Li, Academician of the Royal Academy of Engineering; Zhonglin Wang, the first Chinese to receive the Eni Prize, etc.*

